# Mitochondrial heterogeneity in diseases

**DOI:** 10.1038/s41392-023-01546-w

**Published:** 2023-08-23

**Authors:** Long Chen, Mengnan Zhou, Hao Li, Delin Liu, Peng Liao, Yao Zong, Changqing Zhang, Weiguo Zou, Junjie Gao

**Affiliations:** 1 State Key Laboratory of Cell Biology, CAS Center for Excellence in Molecular Cell Sciences, Shanghai Institute of Biochemistry and Cell Biology, Chinese Academy of Sciences, University of Chinese Academy of Sciences, Shanghai, 200031 China; 2 Department of Pathogenic Biology, School of Basic Medical Science, China Medical University, Shenyang, 110001 China; 3 Department of Orthopaedics, Shanghai Sixth People’s Hospital Affiliated to Shanghai Jiao Tong University School of Medicine, Shanghai, 200233 China; 4 Centre for Orthopaedic Research, Medical School, The University of Western Australia, Nedlands, WA 6009 Australia; 5 Institute of Microsurgery on Extremities, and Department of Orthopedic Surgery, Shanghai Sixth People’s Hospital Affiliated to Shanghai Jiao Tong University School of Medicine, Shanghai, 200233 China; 6 Shanghai Sixth People’s Hospital Fujian, No. 16, Luoshan Section, Jinguang Road, Luoshan Street, Jinjiang City, Quanzhou, Fujian China

**Keywords:** Physiology, Target identification, Microarrays

## Abstract

As key organelles involved in cellular metabolism, mitochondria frequently undergo adaptive changes in morphology, components and functions in response to various environmental stresses and cellular demands. Previous studies of mitochondria research have gradually evolved, from focusing on morphological change analysis to systematic multiomics, thereby revealing the mitochondrial variation between cells or within the mitochondrial population within a single cell. The phenomenon of mitochondrial variation features is defined as mitochondrial heterogeneity. Moreover, mitochondrial heterogeneity has been reported to influence a variety of physiological processes, including tissue homeostasis, tissue repair, immunoregulation, and tumor progression. Here, we comprehensively review the mitochondrial heterogeneity in different tissues under pathological states, involving variant features of mitochondrial DNA, RNA, protein and lipid components. Then, the mechanisms that contribute to mitochondrial heterogeneity are also summarized, such as the mutation of the mitochondrial genome and the import of mitochondrial proteins that result in the heterogeneity of mitochondrial DNA and protein components. Additionally, multiple perspectives are investigated to better comprehend the mysteries of mitochondrial heterogeneity between cells. Finally, we summarize the prospective mitochondrial heterogeneity-targeting therapies in terms of alleviating mitochondrial oxidative damage, reducing mitochondrial carbon stress and enhancing mitochondrial biogenesis to relieve various pathological conditions. The possibility of recent technological advances in targeted mitochondrial gene editing is also discussed.

## Introduction

A mitochondrion is a double-membrane organelle comprising an outer membrane, inner membrane, and matrix.^1^ Mitochondria produce most cellular ATP via the tricarboxylic acid cycle (TCA) and the oxidative respiratory (OXPHOS) chain^2^ and play vital roles in coordinating glucose, lipid, amino acid, and nucleotide metabolism.^3^ In addition, mitochondria are major sources of the cellular production of reactive oxygen species (ROS) and carry numerous redox pathways.^4,5^ Mitochondrial proteins catalyze the biosynthesis of Fe**-**S clusters and one**-**carbon units^6–9^ and maintain mitochondrial morphology via, for example, the formation of mitochondrial cristae, networks, and contacts with other organelles.^10–22^ As semi-independent organelles, mitochondria contain a complete genetic system, which includes the mitochondrial genome (mtDNA) and numerous factors that are crucial for the maintenance and regulation of mtDNA and mitochondrial ribosomes.^23–25^ The proteins encoded by mtDNA are inserted into the OXPHOS chain located on the inner membrane after translation from ribosomes facilitated by oxidase assembly (OXA),^26,27^ and numerous cytosolic signaling cascades are connected to mitochondria under physiological and pathophysiological conditions.^28,29^ The metabolic fitness of mitochondria in response to cellular stress is a measure of mitochondrial quality.^30,31^ Selective degradation of damaged mitochondria through mitophagy has been identified as the classic mechanism of mitochondrial homeostasis maintenance,^32–36^ and other autophagy-independent constituents, such as mitochondrion-derived vesicles (MDVs), and pathways, such as the mitocytosis and mitolysosome exocytosis, have been reported.^37–39^ In contrast to the removal of dysfunctional organelles through mitophagy, the generation of MDVs is a direct outcome of the lateral segregation of cargo into budding vesicles that move along the tubules of functional mitochondria. This process differs from the fission and segregation of mitochondrial fragments.^37^ The mitocytic pathway is intrinsically associated with cell migration and responses to mild mitochondrial stress to prevent the accumulation of damaged mitochondria.^40^ The mitolysosome exocytic pathway seems to be a universal process, and different mechanisms mediate mitochondrial exocytosis, such as CD38-mediated transfer, LC3-dependent exopher trafficking and vacuole-mediated cell extrusion. The lysosome-associated mitolysosome exocytic mechanism may also be vital to mitochondrial quality control.^39^

**Fig. 1:**
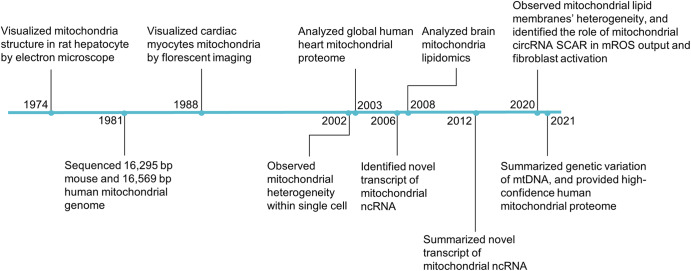
Timeline of mitochondrial heterogeneity research. Key discoveries in the field are highlighted. Abbreviations: ncRNA noncoding RNA, mROS mitochondrial reactive oxygen species

Mitochondrial heterogeneity has been defined as the variation in mitochondrial features between cells or within the mitochondrial population within a single cell. The key initial step in the field of mitochondrial heterogeneity was the reconstruction of electron micrographs that revealed mitochondrial networks in rat hepatocytes in 1974,^41^ and this discovery was reproduced in several cell types, including human endothelial cells and astrocytes,^42^ demonstrating that mitochondrial morphology varies in different cells. In the 1980s, the field was advanced with sequencing of the mouse mitochondrial genome, providing a molecular framework for understanding mtDNA heterogeneity,^43^ culminating in a summary of the extreme genetic variation within mtDNA in 2021.^44^ During the 2000s, studies began to focus on the association between the heterogeneity of mitochondrial proteins, noncoding RNAs (ncRNAs), lipids and the cause of mitochondrial-related disease. The mitochondrial proteome of human heart mitochondria was identified in 2003 and included 615 mitochondrion-associated proteins.^45^ Meanwhile, mitochondrial ncRNAs candidates were systematically identified in mouse mitochondria in 2006,^46^ and a 2008 study into the nonsynaptic (NS) and synaptic (Syn) mitochondrial lipidomes of the mouse brain revealed that lipidomic heterogeneity influenced energy metabolism.^47^ In 2020, a mitochondrial circRNA has been reported to alleviate nonalcoholic steatohepatitis (NASH) by reducing mitochondrial ROS (mROS) output.^48^ More recently, human mitochondrial proteome contains, including 1,134 proteins, was obtained with high confidence (Fig. 1).^49^

In recent decades, mitochondrial purification technology, combined with genomics, transcriptomics, proteomics, and bioinformatics analyses has revealed that mitochondria exhibit a high degree of heterogeneity,^45,50,51^ and some of this heterogeneity can be explained by the varying proportions of mutant and wild-type mtDNA in eukaryotic cells, a state called heteroplasmy. Other causes of heterogeneity can be cell- and tissue-specific differences in mitochondrial ncRNAs and proteins.^52^ This review summarizes the heterogeneous characteristics of mitochondria in the pathological conditions of different tissues.

## Mitochondrial performance diversity

Historically, fluorescence and electron microscopy have been used to observe mitochondria in mammalian cells.^10,13,53^ The statistical analysis of mitochondrial morphology hinges on computer image-processing tools, and using these methods, mitochondria have been observed to be ‘tubelike’ structures that are densely packed in the perinuclear space and that emanate outward from the cell periphery. Mitochondrial morphological statistics are a universal part of mitochondrial research. Here, we summarize the methods and software development processes used to quantify mitochondrial morphology (Table 1). The performance of these processes with mitochondria differs significantly in different cells and tissues, and studies have linked different mitochondrial morphologies with molecular mechanisms.

**Table 1:** Mitochondrial morphology image analysis

Basic parameters	Advanced analysis	Platform	Validation	Reference
Mitochondrial morphological analysis algorithm				
Length/width/count/area/ aspect ratio/number/density/form factor/branch length/point/diameters	Colocalization	ImageJ	CV1-4A	^385^
	Perimeter	Image Pro	Human skin fibroblasts	^386^
	Area-weighted form factor	ImageJ	HeLa. PC12	^387^
	2/3D network-shape integrative analysis	Image Pro	HUVECs	^388^
	Mitochondrial movement in neurons	ImageJ	Neurons	^389^
	TMRM, calcein	Machine learning	pH values Fs	^390^
	Segmentation of mitochondria	Software	Somatosensory cortex	^391^
	Mean network size, mitochondrial footprint,	ImageJ	SH-SY5Y cells	^392^
	ratio of junctions/ends (J/E), tubules/ends	ImageJ	U2OS cells	^11^
	Mitochondrial mass, solidity	ImageJ	Monolayer adherent cells	^393^
	Mitochondrial orientation	Software	Pancreatic tumor cell	^394^
	Mitochondrial segmentation	ilastik	*C. elegans* muscle cells	^395^
	Mitochondrial motion	Software	PC12, H1299, HFF cells	^396^
Mitochondrial classification algorithm				
	Small/swollen globules^2^, straight/twisted/branched tubules^4^, loops^3^	MicroP	CHO cells	^397^
	Punctate^1^. intermediate and filamentous^4^	MATLAB	A2780, OVCA-429, A549, Caco-2 cells	^398^
	Tubular^4^. dount^2^. bolb^1^	GemIdent	BEAS-2B, A549, HT108 cells	^399^
	Network^4^, rod-like^1^, punctate^1^, and large/round^2^	Machine learning	661w cells	^392^
	Punctate^1^, swollen^2^, network^4^	MATLAB	A549 cells	^400^

### Mitochondrial morphology

**Fig. 2:**
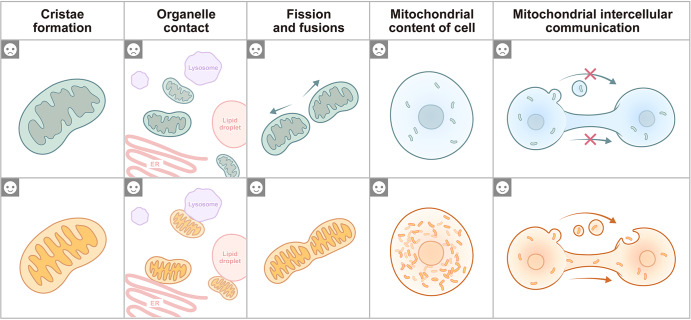
Mitochondrial characteristics under different conditions. Low mitochondrial cristae density is associated with a low cellular energy supply (☹), while a high mitochondrial crista density reflects an adaptation to meet cell energy demands (☺). Mitochondrial contact with many additional organelles, such as the ER, lysosomes and lipid droplets, and the number of contacts mitochondria make with a specific organelle can vary dramatically from only a few contacts to hundreds of contacts per cell. A decrease in the number of mitochondrial connections to other organelles is typically a response to an inefficient metabolic pathway (☹), and in contrast, an increase indicates a response to an active cellular energy metabolic pathway (☺). Mitochondrial fission and fusion are the main pathways of mitochondrial morphology regulation, and under stressful environments, mitochondria are active during mitochondrial fission and produce an increased number of punctate mitochondria (☹), while mitochondrial fusion mediates the formation of mitochondrial networks that adapt to the high energy demands of cells (☺). The content of mitochondria in a cell reflects the intensity of cellular metabolism. Low mitochondrial content in cells is usually associated with low metabolic activity (☹), and high mitochondrial content is associated with high cellular metabolic activity (☺). Intercellular mitochondrial communication is extensive under physiological and pathological conditions, and a low frequency of mitochondrial communication is a response to low cellular adaptation to stressful environments (☹), and in contrast, efficient mitochondrial transfer enhances cellular adaptive capacity (☺)

Mitochondrial morphology varies in different tissues^54^ and depends on the environmental or physiological conditions.^55^ Changes in mitochondrial morphology are mainly due to the fission and fusion of mitochondria,^56–58^ the formation and maintenance of mitochondrial cristae,^53,59^ the contents of the mitochondrial matrix,^60,61^ and connections with other organelles, such as lysosomes and the endoplasmic reticulum (ER) (Fig. 2).^62,63^ Mitochondrial fission and fusion occur simultaneously and continuously during metabolic processes in eukaryotic cells.^64,65^ Fission can produce daughter mitochondria and aids in quality control by allowing damaged mitochondria to be removed and by triggering apoptosis in response to high levels of cellular stress.^66,67^ The coordination of cytoplasmic, cytoskeletal, and organellar components is required for fission.^68^ In yeast, the dynamin-related GTPase Dnm1 has been shown to localize to mitochondrial fission sites via recruitment by adaptor proteins (Fis1, Mdv1 and Caf4) to regulate mitochondrial fission.^69^ The mammalian homolog DRP1 is affected by adaptor proteins (Mff, MiD49, and MiD51) rather than mammalian Fis1, which is involved in mitophagy instead of being implicated as an adaptor protein.^67,70^ Fusion is a supplementary mitochondrial quality control mechanism that contributes to stress relief by mixing contents of partially damaged mitochondria.^71^ Fusion can alter mitochondrial function in response to cytosolic signaling. MFN1/2 are mammalian effectors of outer mitochondrial fusion, while OPA1, another key player, is involved in sculpting the inner mitochondrial membrane.^72–75^ Recent studies have suggested that mitochondrial fission and fusion rates change in response to energetically demanding cellular behaviors^76^ and extreme conditions (e.g., disease, parasitic infection, and starvation).^71^ Genetic and environmental factors that affect mitochondrial fission and fusion can also directly impact tissue performance, such as embryonic development,^72^ organ lesion repair,^77–83^ and tumorigenesis.^84^

Mitochondrial cristae remodeling is closely related to mitochondrial respiration, and the electron transport system (ETS) embedded within the mitochondrial cristae directly impacts ATP production.^85^ Researchers using cryoelectronic tomography have revealed that an increase in energy production is accompanied by an increase in the formation of mitochondrial cristae. In living cells, researchers recently found that OPA1, Yme1L, MICOS, and Sam50, along with the newly identified cristae regulator ATAD3A, controlled mitochondrial cristae dynamics.^10,86–88^ Genetic and environmental factors that affect mitochondrial cristae formation and maintenance can also impact animal biology, such as the maintenance of constant body temperature,^53^ lesion repair in multiple organs^89,90^ and immune responses to the tumor.^91^ Cold stimulation of brown adipose tissue (BAT) led to enhanced cristae formation, which was attributed to the interorganelle PERK-OGT-TOM70 axis increasing cell respiration through mitochondrial protein import and subsequent cristae formation.^92^ Notably, PD-1 signaling promotes the exhaustion of activated T cells. A study discovered a reduction in the number and length of mitochondrial cristae in PD-1-stimulated cells.^91^

Mitochondrial content measured by the number of the mitochondrial genome copies has been shown to be different in the mitochondria of different mammalian organs.^93^ The contents of intercellular mitochondria is higher in metabolically active cell types than that in less metabolically active cell types.^93^ For instance, the human myocardium is composed of 23% mitochondria by volume density.^94,95^ Normal liver cells contain approximately 21% mitochondria.^96,97^ Mitochondrial content in human skeletal muscle varies from 4% to 15%.^98^ White adipose tissue (WAT) present with fewer and smaller mitochondria than BAT.^99^ And the number of mitochondria diminishes during tissue regeneration and cancerogenesis.^100–102^

### Mitochondrial distribution during mitosis

**Fig. 3:**
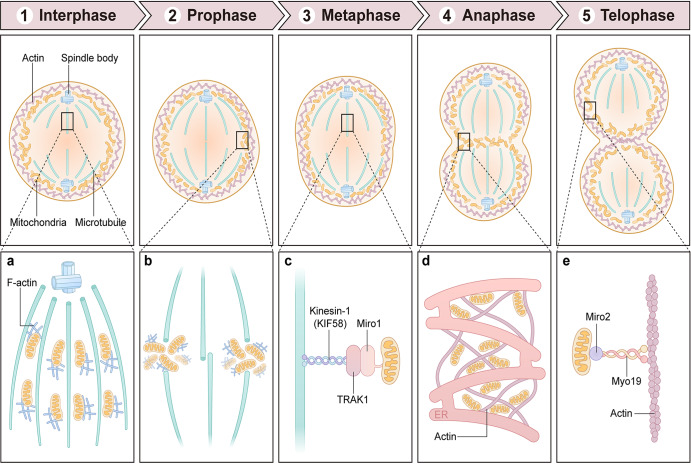
Mitochondrial distribution during mitosis. During interphase, the mitochondrial network is evenly distributed in the cytoplasm. During prophase, the mitochondrial network is crumpled and primarily located in the perinuclear area, where punctate mitochondria are apparent. During metaphase, mitochondria move to the equatorial plane in the midline of a cell at right angles to the axis. During anaphase, mitochondria move to the opposite ends of a cell. During telophase, the mitochondrial network is re-formed and grouped at either pole of a cell. **a** The association of mitochondria with cytoplasmic F-actin may promote mitochondrial distribution during mitosis. **b** Mitochondrial delivery on microtubules may dock to actin in the cleavage furrow. **c** Miro-1 is required for transporting mitochondria to the plus ends of microtubules at the cleavage furrow via interaction with KIF5B. **d** Close association between mitochondria and both ER sheets and actin cables may promote mitochondrial distribution during mitosis. **e** Myo19 is localized to mitochondria and acted as a novel actin-based motor that controls mitochondrial distribution during mitosis

Mitochondrial distribution is usually associated with the cytoskeleton and centrioles.^103,104^ It depends on the direction of the diffusion currents within cells and is related to the submicroscopic organization of the cytoplasmic matrix and vacuolar system (Fig. 3).^105^ The ER, which forms a scaffold with mitochondria, is organized into a dense meshwork of subcortical actin cables assembled throughout the cytoplasm of mitotic cells.^105^ As they are co-oriented with nearby cables, mitochondria are positioned within meshwork pores. Cytochalasin D (CytoD)- or latrunculin A (LatA)-induced meshwork removal caused mitochondria to aggregate, disrupting their symmetrical arrangement and cell division.^105^

## Mitochondrial components

**Fig. 4:**
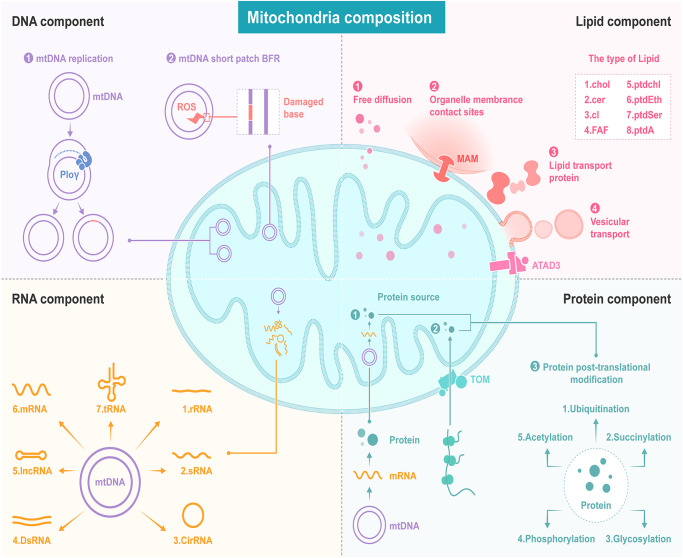
The heterogeneity of mitochondrial components. DNA component: The diversity of the mitochondrial genome arises from base-pair mismatches during the replication of the genome and base mutations after mtDNA short-patch base excision repair (BER). RNA component: Transcripts from the mitochondrial genome include rRNAs, sRNAs, CircRNAs, dsRNAs, IncRNAs, mRNAs and tRNAs. The protein component diversity of the mitochondrial proteome arises from the two main protein resources, the nucleic coding protein import pathway and the mtDNA translation pathway. In addition, mitochondrial proteins are altered via a complex posttranslational modification mechanism. The heterogeneity of mitochondrial lipids is a result of four related lipid transport pathways, including free diffusion, organelle membrane contact, lipid transport proteins and vesicular transport

Mitochondrial composition shows a high degree of heterogeneity. Here, we summarize the differences among mitochondrial genomes, transcriptomes, proteomes, and lipidomes in cells and describe the related underlying molecular mechanisms (Fig. 4).

### Mitochondrial DNA

The mitochondrial genome forms a compact, double-stranded circle comprising 16.5 kb with a cytosine-rich light (L) chain and a guanine-rich heavy (H) chain.^106^ Contrary to the long-held view that most humans harbor only identical mitochondrial genomes, massively parallel deep resequencing has revealed unanticipated extreme genetic variation within mtDNA at multiple levels.^44^ Multiple mtDNA genotypes are present at both the cellular and organelle levels^107^ and can be inherited from both parents.^108,109^ In contrast to the nuclear genome, mtDNA is not supported by histones to protect them against ROS damage.^110^ Recent studies have shown that the replication sites of mtDNA are physically close to the oxidative respiratory chain, and the production of ROS during oxidative respiration makes mtDNA more susceptible to damage.^111^ On the other hand, most age-related mtDNA mutations are thought to be due to errors in mtDNA replication,^112^ which has been confirmed to exert a far greater impact than ROS-mediated mtDNA variation.^113^ The mechanisms of mtDNA escape from mitochondria to intracellular or extracellular compartments depend on pores formed by Bax and Bak, VDAC oligomers, and mitochondria permeability transition pores (mPTPs).^114^ The mtDNA continuously and independently replicates during the cell cycle with a half-life that varies from days to weeks depending on the cell type. For example, it is 8–12 days for epithelial cells and 20-25 days for neurons.^115,116^ Although the polymerase-γ, which is critical for mtDNA replication, shows high fidelity,^117^ the large number of mtDNA replication cycles that are required over the lifetime of a cell inevitably induces base-pair substitution errors. The origins of human mtDNA variation and its relevance for human diseases, including cancer, neurodegenerative diseases and aging, have been studied. It is commonly assumed that mtDNA is mutated at a faster rate than nuclear DNA (nDNA) in eukaryotes.^118^

### Mitochondrial genome-generated noncoding RNAs

The complexity of transcripts derived from mitochondria is beginning to be understood, and advances in deep-sequencing technology has supported findings of ncRNAs encoded in mitochondria.^119^ Various ncRNAs are derived from mitochondria, and the mitochondrial genome gives rise to hundreds of circRNAs,^48,120,121^ at least eight lncRNAs,^122^ a few dsRNAs^123^ and various small RNAs.^51^ Whether moving into the cell membrane or the nucleus or remaining in the mitochondria, these ncRNAs carry out a variety of biological tasks. Global transcriptome profiling revealed that in the human left ventricle, a relatively high abundance (71%) of lncRNAs is encoded by the mitochondrial genome.^124^ Using a PacBio full-length third-generation sequencing transcriptome dataset, researchers identified two polycistronic transcripts, namely, hsa-MDL1 (mitochondrial D-loop 1) and hsa-MDL1AS (mitochondrial D-loop 1 antisense), generated from a region covering the tRNA^Pro^ gene and the full length of the human D-loop region.^125^ Pan RNA-seq analysis also revealed that the 5’- and 3’-end small RNAs of MDL1 and MDL1AS were ubiquitous.^125^ Interestingly, highly unstable mitochondrial double-stranded RNA (mt-dsRNA) has been found in HeLa cells.^123^ Bax-Bak-dependent release of mt-dsRNAs into the cytoplasm triggered the upregulation of interferon-stimulated genes and the activation of innate immune defenses mediated through MDA5-MAVS.^123^ Second-generation sequencing of mitochondrial RNAs has led to the identification of mammalian mitochondrial genome-encoded circRNAs.^120^ When investigating the role of mitochondrion-localized circRNAs during metaflammation, researchers showed that one circRNA, named SCAR, bound directly to ATP5B. The interaction of ATP5B and SCAR abrogated mPTP by blocking induced by the cyclophilin D-mPTP interaction and therefore inhibited mROS production.^48^ Another highly expressed mecciRNA, mc-COX2, a sense RNA encoded by the COX2 locus, was found in the plasma exosomes of chronic lymphocytic leukemia (CLL) patients. The prognosis of CLL was closely correlated with mc-COX2 level, with higher expression levels of mc-COX2 seemingly promoting cell proliferation and protecting cells from apoptosis. Notably, in a comprehensive description of murine and human mitochondrial transcriptomes,^51^ thousands of small noncoding RNAs (sncRNAs) aligned to the mitochondrial genome at positions corresponding to 16S rRNA, tRNA, and mRNA.

### Mitochondrial proteome and protein posttranslational modifications (PTMs)

At least 1100 proteins have been identified with high confidence as members of the mitochondrial proteome.^49^ However, among these proteins, only 13 proteins are encoded by mtDNA in mammalian cells, included ND1, ND2, ND3, ND4, ND4L, ND5, ND6, CO1, CO2, CO3, ATP6, ATP8 and Cyt b,^110^ therefore, the remaining 99% must be imported into mitochondria after precursors are synthesized on cytosolic ribosomes.^126^ With the advancement of mitochondrial isolation technology, the mitochondrial proteome has become a hotspot for organelle proteome research.^127,128^ The MitoCarta database, a collection of manually annotated mitochondrial proteins with submitochondrial localizations and functions, is a continually updated community resource used for investigating mitochondrial biology.^129^

The cell-specific mitochondrial proteome composition is associated with the metabolism characterized.^130^ Large-scale proteomic surveys have provided valuable molecular insights into tissue diversity and indicated that mitochondria obtained from distinct organs share approximately 75% of proteins.^131^ Furthermore, the use for mitochondrial proteome data has been gradually evolved from identifying differences in mitochondrial content to identifying differences in mitochondrial protein PTMs and characterizing the dynamics of protein interactions. Numerous mitochondrial protein PTMs, such as phosphorylation, acetylation, methylation, ubiquitination, SUMOylation, glycosylation, and nitrosylation, have been reported.^132^ Pioneering research has revealed that reversible phosphorylation of liver mitochondrial proteins controls ketogenesis.^133^ Systematic phosphoproteomes of rat liver, heart and skeletal muscle showed that phosphoproteins were involved in amino acid and fatty acid metabolism in liver mitochondria, whereas heart and skeletal muscle were enriched for phosphoproteins involved in energy metabolism.^134^ Myocardial acetylproteomics demonstrated extensive mitochondrial protein lysine hyperacetylation in the early stages.^135^ Proteomics techniques have thus revealed that mitochondrial protein PTMs vary between cells.

The underlying mechanism of mitochondrial heterogeneity is mainly due to mitochondrial protein import and the mitochondrial protein modification pathway. The mitochondrial proteins encoded by nuclear genes are synthesized on cytosolic ribosomes and imported into mitochondria through signal-targeting peptides and pathways through which precursor proteins are imported into mitochondria.^3^ This presequence pathway is typically recognized by translocase of the outer membrane (TOM) and translocase of the inner membrane (TIM).^3^ Studies on the TOM complex, consisting of receptor proteins (TOM70, TOM20 and TOM22) and a pore-forming protein (TOM40), revealed that the reversible phosphorylation of TOM complexes contributes to the formation of supercomplexes and controls the activity of distinct import routes.^136^ Moreover, two of these presequence translocases are differentially distributed across tissues. One form includes the stably expressed housekeeping subunit TIM17B, and the other form includes the stress-regulated subunit TIM17A,^137^ suggesting that additional regulatory mechanisms contribute to mitochondrial heterogeneity in multicellular organisms. The mechanism of mitochondrial protein PTM heterogeneity may be due to mitochondrion-localized protein modification enzymes. Sirtuin 3, an NAD(+)-dependent protein deacetylase, has been shown to be located in mitochondria and regulates the acetylation levels of mitochondrial proteins.^138^ A kinase prediction showed important roles for PKA and PKC at the phosphorylation sites of mitochondrial proteins.^134^

### Mitochondrial lipids

Mitochondria are unique organelles for studying membrane biochemistry because their functionality depends on a coordinated supply of proteins and lipids. Most phospholipids, sterols, sphingolipids, and neutral lipids are synthesized within the ER, but mitochondria contribute to the cellular synthesis of phosphatidylethanolamine (PtdEth).^139^ In recent decades, mitochondrial lipidomic analysis has revealed that mitochondria from various organs carry phosphatidylcholine and phosphatidylethanolamine comprising 5–30% of total phospholipids.^140,141^ Another subcellular organelle lipidomic study of living cells revealed sophisticated lipid dynamics during mitochondrial cristae dissociation at different stages.^142^ Despite the gradual recognition of mitochondrial lipid heterogeneity, the molecular mechanisms associated with the regulation of mitochondrial lipids are not clearly understood. Mitochondria play central roles in the catabolic degradation of fatty acids (β-oxidation) and, to some degree, in fatty acid synthesis, which involves frequent communication with other cellular compartments, such as the ER and peroxisomes.^59,76^ ER-mitochondrion contact sites, which are called mitochondrion-associated membranes (MAMs), allow the exchange of lipids between both organelles,^143^ and lipid droplets derived from the ER are storage reservoirs for sterols and fatty acids in the form of triacylglycerols (TAGs) and steryl esters.^144,145^ Hence, intercellular lipid transport from other organelles to mitochondria is clearly important. Similarly, mitochondrial activities depend on lipid exchange between the IMM and outer mitochondrial membrane (OMM).^59,146^

## Intracellular mitochondrial heterogeneity

Studies have revealed intracellular mitochondrial heterogeneity in mitochondrial components, mitochondrial morphology and mitochondrial function in cells that depend on the mitochondrial redox state, membrane potential, respiratory activity and ROS production.^147–150^ Taking advantage of high-resolution techniques that can be used to identify all types of mtDNA structural variations and single-nucleotide variations (SNVs) in a single cell, intracellular heterogeneity of mtDNA in single neuron mouse cells or human peripheral blood mononuclear cells, hematological cancers, fibroblasts and tumor cell lines has been discovered.^106^ The most common mtDNA mutation, 8344A>G, has been observed in cells exhibiting a broad range of heteroplasmy (from 0% to 100%).^106^

Regarding mitochondrial morphology and function, confocal microscopy led to the a distinction being made in mitochondrial subpopulations of specific cell regions based on the immunostaining of mitochondria-specific markers.^42^ In neurons, distinguishable morphological and compositional variation was found between neuron synaptic mitochondria (sMito) and nonsynaptic mitochondria (nsMito).^151–155^ Proteomic and enzymatic characteristics of the synaptic mitochondrial subpopulation revealed that the levels of 22 proteins were significantly higher and those of 34 proteins were significantly lower in sMito than in nsMito. These proteins included the mitochondrial ROS clearance-related protein-superoxide dismutase [Mn] (SOD2), TCA-related protein-isocitrate dehydrogenase subunit alpha (IDH3a), aconitate hydratase (ACO2), and ATP-forming β subunit of succinyl-CoA ligase (SuclA2). The OXPHOS-related protein NADH dehydrogenase includes ubiquinone iron-sulfur protein 8 (Ndufs8) and cytochrome c oxidase subunit 5A (Cox5a).^156^ Further study demonstrated that the 3 most distinct clusters identified between sMito and nsMito by proteomic expression profiling were associated with glycolysis, OXPHOS and inner membrane bioenergetic complexes.^157^

Intracellular mitochondrial subpopulations may exhibit different responses to substrates and may vary in their sensitivity to deleterious stress.^158,159^ In neurons, sMito exhibited enhanced respiration activity and increased vulnerability to Ca^2+^ overload and oxidative damage compared to nsMito.^153,156^ The enhanced respiration activity of sMito ensures an energy supply for the extension and branching of neuronal axons and dendrites, while increased vulnerability to Ca^2+^ overload and oxidative damage compared to nsMito makes neuronal axons more vulnerable to oxidative stress. Studies have also revealed that energy shortage and accumulation of Ca^2+^ and ROS at individual synapses may lead to synaptic loss, which is an early sign of certain neurodegenerative diseases such as Alzheimer’s disease (AD), amyotrophic lateral sclerosis (ALS), and Parkinson’s disease (PD).^160^

Because of the approach established to isolated peridroplet mitochondria (PDM), mitochondrial heterogeneity has also been demonstrated in adipose cells. PDM show unique morphological and enzymatic features compared to cytoplasmic mitochondria.^161^ The PDM subpopulation of adipose cells has been shown to exhibit enhanced bioenergetic capacity, low fatty acid oxidation capacity and lipid droplet expansion support by providing ATP for triacylglyceride synthesis.^161^ These intracellular mitochondrial subpopulation characteristics provide an explanation for the capacity of mitochondria within individual cells to be simultaneously involved in different metabolic pathways.

## Mitochondrial heterogeneity under pathological conditions

**Fig. 5:**
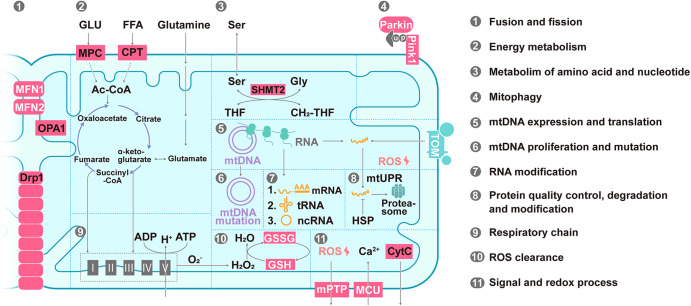
Summary of mitochondrial functions. (1) Fusion and fission. (2) Energy metabolism. (3) Metabolism of amino acids and nucleotides. (4) Mitophagy. (5) mtDNA expression and translation. (6) mtDNA proliferation and mutation. (7) RNA posttranscriptional processing. (8) Protein quality control, degradation and modification. (9) The respiratory chain. (10) ROS clearance. (11) Signaling and the redox process. Abbreviations: MPC mitochondrial pyruvate carrier, CPT carnitine palmitoyltransferase, GLU glucose, FFA free fatty acid, Ser serine, Gly glycine, THF tetrahydrofolate, CH_2_-THF 5,10-methylene-THF, mtDNA mitochondrial DNA, mRNA messenger RNA, tRNA transfer RNA, ncRNA noncoding RNA, mtUPR mitochondrial unfolded protein response, HSP heat shock protein, ROS reactive oxygen species, CytC cytochrome c, GSH glutathione and GSSG glutathione oxidized

**Fig. 6:**
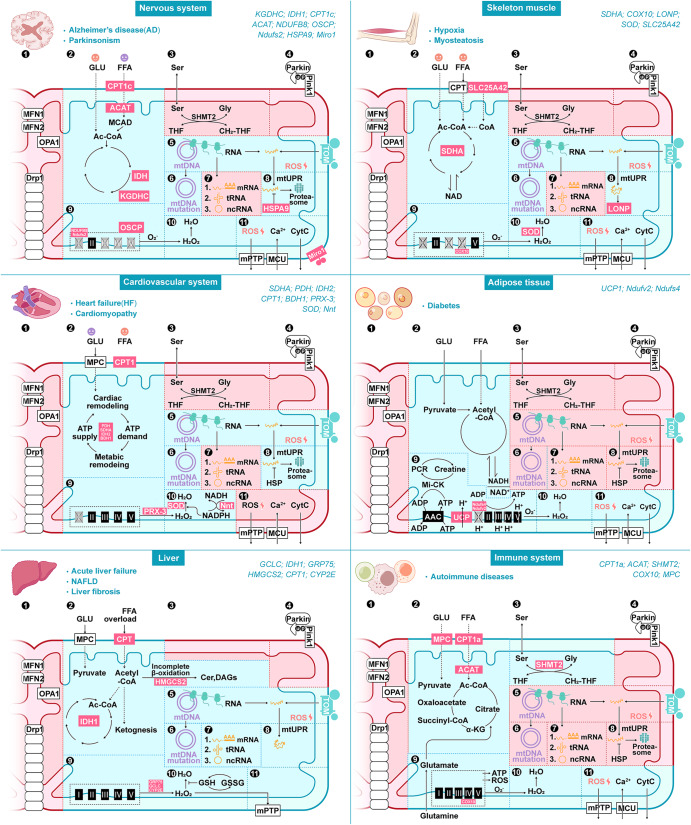
Comparison of mitochondrial heterogeneity in tissues. Pathways with significant differences in the mitochondrial proteome of tissue in pathological states are marked with cyan colors, and no significant differences in mitochondrial proteomic results or mitochondrial pathways with changes that were not based on mitochondrial proteomic results are marked with pink colors. Based on the research of mitochondrial-targeted transgenic mice model, we summarized the relationship between mitochondrial protein and disease pathological states, all related proteins are marked with magenta. Brain pathology state, Alzheimer’s disease (AD) and Parkinson’s disease (PD), mitochondrial function associated with (2) energy metabolism; (5) mtDNA expression and translation; (6) mtDNA proliferation and mutation; (8) protein quality control, degradation and modification; (9) the respiratory chain; (10) ROS clearance; (11) signaling and the redox process. Skeletal muscle pathology state, hypoxia and myosteatosis, mitochondrial function associated with (2) energy metabolism; (5) mtDNA expression and translation; (6) mtDNA proliferation and mutation; (8) protein quality control, degradation and modification; (9) the respiratory chain; (10), ROS clearance; (11) signaling and the redox process. Cardiovascular pathology states, heart failure (HF) and cardiomyopathy, mitochondrial function associated with (2) energy metabolism; (5) mtDNA expression and translation; (6) mtDNA proliferation and mutation; (8) protein quality control, degradation and modification; (9) the respiratory chain; (10) ROS clearance; (11) signaling and the redox process. Adipose tissue, mitochondrial function associated with (2) energy metabolism; (9) thermogenesis; (10) ROS clearance; (11) signaling and the redox process. The liver, mitochondrial function associated with (2) energy metabolism; (5) mtDNA expression and translation; (6) mtDNA proliferation and mutation; (7) RNA posttranscriptional processing; (8) protein quality control, degradation and modification; (9) the respiratory chain; (10) ROS clearance; (11) signaling and the redox process. Immune system mitochondrial function associated with (2) energy metabolism; (3) metabolism of amino acid and nucleotides; (9) the respiratory chain; (10) ROS clearance; (11) signaling and the redox process. Abbreviations: CPT1c Carnitine Palmitoyltransferase 1C, ACAT acetyl-CoA acetyltransferase 1, IDH1 isocitrate dehydrogenase 1, KGDHC α-ketoglutarate dehydrogenase complex, OSCP oligomycin sensitivity-conferring protein, NDUFB8 NADH:ubiquinone oxidoreductase subunit B8, Ndufs2 NADH dehydrogenase [ubiquinone] iron-sulfur protein 2, HSPA9 Mortalin, Miro1 Mitochondrial Rho-GTPase, SDHA Succinate dehydrogenase complex, subunit A, PDH pyruvate dehydrogenase, BDH1 3OH-Butyrate dehydrogenase, type1, PRX-3 Peroxiredoxin 3, SOD superoxide dismutase, Nnt nicotinamide nucleotide transhydrogenase, GCLC glutamate cysteine ligase catalytic, GRP75 Glucose-regulated protein 75 kDa, HMGCS2 3-Hydroxy-3-methylglutaryl CoA synthase 2, CYP2E cytochrome P450-2E1, COX10 Cytochrome c oxidase assembly homolog 10, LONP Lon protease homolog, SLC25A42 Mitochondrial coenzyme A (CoA) transporter, UCP1 uncoupling protein 1, Ndufv2 NADH dehydrogenase [ubiquinone] Flavoprotein 2, Ndufs4 NADH dehydrogenase [ubiquinone] iron-sulfur protein 4, SHMT2 Serine hydroxymethyl transferase 2 and MPC mitochondrial pyruvate carrier

In addition to pathways for energy metabolism, such as the TCA cycle, OXPHOS and fatty acid oxidation (FAO), several other pathways are activated in mitochondria, such as mtDNA transcription and translation; amino acid, lipid, and nucleotide metabolism; calcium homeostasis; apoptosis signaling; and redox process pathways (Fig. 5).^3^ Annotations of the mitochondrial genome and proteome have allowed for in-depth studies of the biochemical function, evolutionary history, and diversity of mitochondria in cells and tissues (Table 2). More than 150 mtDNA mutations have been associated with maternally inherited syndromes (Table 2).^162^ Recent research has been aimed to separate pathogenic from benign variants and to create technical tools for the precise editing of the mitochondrial genome.^163,164^ The majority (31%) of various mitochondrial proteins examined via mitochondrial proteomic analyses exhibit a function related to metabolism.^49^ The abundance of mitochondrial proteins involved in quality control, signaling, regulatory functions, and membrane dynamics is typically low, and 20% of proteins with low abundance perform unannotated functions and may involve substoichiometric regulatory factors.^49^ Many other orphan mitochondrial proteins that lack robust functional characterization have been suggested to play biological roles.^165^ An intensive characterization of the proteome has led to the identification of many mitochondrial proteins that appear to be involved in the reversible PTMs of proteins, such as phosphorylation and acetylation,^133,135,166^ suggesting a complex signaling network within the organelle. Notably, the current understanding of proteomic signatures in various organs under pathological conditions are important to summarize, as pioneering efforts in recent decades have established the core protein components of mitochondria.^167^ Genetic and pharmacological mouse models to which mitochondrial proteins can be targeted have provided the basis for a more detailed understanding of mitochondrial heterogeneity in different cellular, tissue and pathological states (Table 3 and Fig. 6), which may be important references for identifying mitochondrial targets for disease therapy.

**Table 2:** Disease-related mitochondrial omics sequencing data summary

Organ	Disease	Experimental method	Reference
Nervous system	Mitochondrial encephalomyopathy	mtDNA sequencing	^170,171^
	Cerebral metabolic abnormalities	mtDNA sequencing	^172^
	Parkinson’s disease	mtDNA sequencing. Proteome	^174,175^
	Frontotemporal lobar degeneration	mtDNA sequencing	^401^
	Neurodegeneration	Proteome	^130^
	Alzheimer’s disease	Proteome. Interatomic analysis	^195,402^
	Amyotrophic lateral sclerosis	Interatomic analysis	^403^
	Spinal muscular atrophy	Proteome	^176^
	Neurodegenerative disease	Proteome	^177^
Cardiovascular system	Heart failure	Acetylproteome. Proteome	^135,216,217,404^
	Myopathy and progressive external ophthalmoplegia	Genome sequencing	^405^
	Mitochondrial cardiomyopathy	Genome sequencing	^406^
	Leigh syndrome	mtDNA sequencing	^407^
	Cardiovascular diseases	mtDNA sequencing	^215^
	Cardiac hypertrophy and heart failure	Proteome	^408^
	Atrial fibrillation	Proteome	^409^
	Proteome comparison of human hearts	Proteome	^410^
Liver	Hepatocellular carcinoma	mtDNA sequencing	^50,230^
	Nonalcoholic fatty liver disease	Proteome	^232,233,411,412^
	Aging and the development of liver diseases	Proteome	^413^
	Cholangiocarcinoma	Proteome	^414^
	Liver fibrosis	Proteome	^415^
Skeletal muscle	Aging	mtDNA sequencing	^273^
	Myopathy	mtDNA sequencing	^271^
	Obesity and T2D, muscle IR	Proteome	^270,274,277–279,416^
	Aging	Proteome	^267,269,280,417^
	Myosteatosis	Proteome	^283,418^
Adipose	Aging	Proteome	^291,292,299^
	Diabetes	Proteome	^289,290,298^
Immune cell	Immune cells	mtDNA sequencing	^315^
	Innate immune-monocyte-septic shock	Proteome	^316^
	Innate immune-macrophage-bacterial resistance	Proteome	^319^
	Innate immune-natural killer cells-	Proteome	^419^
	adaptive immunity-T-cells	Proteome	^31,320,321^
Cancer	Kidney, colorectal and thyroid cancers	mtDNA sequencing	^420^
	Nasopharyngeal carcinoma	Proteome	^333^
	Human ovarian cancer	Proteome	^334,335^

**Table 3:** Disease-related mitochondrial proteins

Disease	Regulation protein	Functional distribution	Reference
Neuron system			
Alzheimer’s disease	CPT1^P.G^, BDH1, SCOT, ACAT1	FAO	^197,201–203^
	IDH1, KGDHC^P^	TCA cycle	^178,180,421^
	COX5A, NDUFB8^G^, OSCP^G^, UQCRC2^G^	OXPHOS	^189–194,422–424^
	HSPA9, HSP60^P^, CLPP^G^	UPR^mt^	^208,209,425^
Parkinson’s disease	KGDHC^G^	TCA cycle	^179,426^
	Miro1^G^	Mitochondria synaptic transmission, mitophagy	^427–432^
	Ndufs2^G^	OXPHOS	^196^
Cardiovascular system			
Heart failure	NDUFAB1^G^, NDUFV7^G^	OXPHOS	^433,434^
	PDH^G^, SDHA^G^, IDH2^G^	TCA cycle	^135,221,222^
	CPT1^G^, BDH1^G^	FAO	^223,224,435^
	SOD2^G^, PRX-3^G^, p66Shc^G^	ROS reduction	^216,226,436,437^
	MPC^G^	Pyruvate transport	^438^
Liver disease			
Acute liver failure	GSH^G^	ROS reduction	^236,237^
	HSP10, HSP60^P^	UPR	^234,240,241^
	GRP75^G^	Transporters and channels	^234,243^
	IDH1^G^	TCA cycle	^239^
NAFLD	CYP2E1^P.G^	ROS generation	^256,257^
	SOD1, SOD2, Gclc^P^, Gclm^P^, Gpx1^P.G^, GSH^G^	ROS reduction	^258–262^
	NQO1^G^	Redox process	^439,440^
	ACAT, ACAC/ACC CPT1α^P^, CPT2α, BDH2^G^	FAO	^253,254,441^
	MPC1, MPC2^G^, SLC25A11^P^	Transport related to TCA cycle	^255,442,443^
	HMGCS2^G^	Ketogenesis	^444–447^
	PHGDH^G^, PSAT1, PSPH	SSP	^255,448^
Liver fibrosis	p66Shc^P.G^	ROS generation	^449–451^
	SOD2^P^, GSH^P^	ROS reduction	^420,452–455^
	Hmgcs2^P.G^	Ketone body production	^444,446,456^
	IDH1^P.G^	TCA cycle, AA utilization	^457,458^
Skeletal muscle			
Myopathy	SDHA^G^	TCA cycle	^459–462^
	CACT, CPT2^P^, ACADVL	FAO	^463–465^
	COX10^P.G^, NDUFB8, UQCRC1	OXPHOS	^462,466–469^
	LONP1^G^, HSP70	UPR^mt^	^470,471^
	SOD2^G^	ROS reduction	^472^
	SLC25A42^G^	Coenzyme A import	^473^
Adipose			
Diabetes	UCP1^P.G^	Thermogenesis	^301–305,474^
	Ndufv2^G^, Ndufs4^G^	OXPHOS	^306,307^
	MnSOD^G^	ROS reduction	^308,475^
Immune cell			
Adaptive immunity	CPT1a^G^, ACAT^P^	FAO	^476–478^
	SHMT2^G^	One-carbon	^31,321^
	COX10^G^	OXPHOS	^320^
	MPC^P.G^	Pyruvate import	^323^

^P^Indicates proteins that have been studied pharmacologically; ^G^ indicates proteins that have been studied genetically

*CPT-1* Carnitine palmitoyltransferase-1, *CPT1α* Carnitine palmitoyltransferase-1α, *BDH1* 3OH-Butyrate dehydrogenase, type1, *SCOT* Succinyl-CoA,3-ketoacid CoA transferase, *ACAT1* Acetyl-CoA acetyltransferase 1, *IDH1* Isocitrate dehydrogenase (NADP( + )) 1, *KGDHC* α-Ketoglutarate dehydrogenase complex, *COX5A* Cytochrome c oxidase subunit 5A, *NDUFB8* NADH,ubiquinone oxidoreductase subunit B8, *OSCP* Oligomycin sensitivity-conferring protein, *UQCRC2* Ubiquinol-cytochrome c reductase core protein 2, *HSPA9* Mortalin, *Hsp60* chaperonin 60, *CLPP* Caseinolytic protease P, *MCAD* Medium-chain acyl-CoA dehydrogenase, *Miro1* Mitochondrial Rho-GTPase, *Ndufs2* NADH dehydrogenase [ubiquinone] iron-sulfur protein 2, *NDUFAB1* NADH,ubiquinone oxidoreductase subunit AB1, *NDUFV7* NADH dehydrogenase [ubiquinone] flavoprotein 1, *PDH* Pyruvate dehydrogenase, *SDHA* Succinate dehydrogenase complex, subunit A, *SOD2* Superoxide dismutase 2, *PRX-3* Peroxiredoxin 3, *p66Shc* Src homology 2 domain-containing transforming protein C1, *MPC* Mitochondrial pyruvate carrier, *GSH* Glutathione, *HSP10* chaperonin 10, *GRP75* Glucose-regulated protein 75 kDa, *CYP2E1* Cytochrome P450-2E1, *SOD1* Superoxide dismutase 1, *Gclc* Cysteine ligase catalytic subunit, *Gclm* Glutamate-cysteine ligase modifier subunit, *GPX 1* Glutathione peroxidase 1, *NQO1* NAD(P)H,Quinone oxidoreductase 1, *HMGCS2* 3-Hydroxy-3-methylglutaryl CoA synthase 2, *ACAT* Acetyl-CoA acetyltransferase, *ACAC/ACC* Acetyl-CoA carboxylase, *SLC25A11* Oxoglutarate carrier, *PHGDH* Phosphoglycerate dehydrogenase, *PSAT1* Phosphoserine aminotransferase 1, *PSPH* Phosphoserine phosphatase, *SLC25A42* Mitochondrial coenzyme A (CoA) transporter, *ACADVL* Acyl-CoA dehydrogenase very long chain, *UQCRC1* Cytochrome b-c1 complex subunit 1, *LONP1* Lon protease homolog, *HSP70* Chaperonin 70, *UCP1* Uncoupling protein 1, *Ndufv2* NADH dehydrogenase [ubiquinone] Flavoprotein 2, *Ndufs4* NADH dehydrogenase [ubiquinone] iron-sulfur protein 4, *MnSOD* Manganese superoxide dismutase, *SHMT2* Serine hydroxymethyl transferase 2, *COX10* Cytochrome c oxidase assembly homolog 10, *UPRmt* mitochondrial unfolding protein response, *SSP* Serine synthesis pathway, *AA utilization* Amino acid utilization

### The nervous system

The brain is a vital organ that consumes massive amounts of energy and depends upon glucose as its main source of energy,^168^ and a close connection between glucose metabolism and mitochondrial function is critical to brain physiology.^169^ Due to the disruption of mitochondrial fusion and fission, mitochondrial morphology heterogeneity has emerged in several neurodegenerative diseases (AD and PD).^65^ Strong evidence has implicated increased mitochondrial component heterogeneity as a central pathological mechanism underpinning neurodegenerative diseases. Multiomics sequencing of neurodegenerative tissues has indicated that abnormalities in the mitochondrial genome and proteome are significantly associated with neuropathological status (Table 2). Major deletions or mutations in the mitochondrial genome of brain tissue have been associated with neurodegenerative diseases, and mtDNA point mutations have been linked to an insufficient energy supply in neurons.^170–172^ Further research revealed accumulated mtDNA mutations during aging in a mtDNA polymerase (POLG) mutant mouse model and worsened neurodegeneration in an AD mouse model established with mice bred with POLG mutant mice.^173^ Additionally, mutation accumulation is present in human neurodegenerative diseases. For example, PD patients with primary substantia nigra (SN) neuron mitochondrial defects presented with POLG mutations,^174^ and substantial loss in the number of SN neurons was observed in patients with POLG mutations, suggesting a correlation between mtDNA mutation accumulation and brain pathology.^174^ Proteome analysis has revealed the important roles of mitochondria in brain tissue. For example, the proteome of neural stem cells (NSCs) in the subventricular zone (SVZ) from PD patients led to the identification of numerous proteins implicated in mitochondrial activity.^175^ Spinal muscular atrophy (SMA) is caused by a reduction in survival motor neurons (SMNs) due to disruption in mitochondrion-associated energy-generating functions.^176^ In addition to mitochondrial energy synthesis in neurons, mitochondrial proteostasis plays an important role in regulating mouse brain autophagic vesicle formation.^177^ The proteome in multiple neurodegenerative diseases exhibits mitochondrial molecular diversity among three major cerebellar cell types (Purkinje cells, granule cells and astrocytes),^130^ supporting a correlation between mitochondrial metabolism and the progression of brain diseases, including AD and PD (Fig. 6 and Table 3).

The TCA cycle, OXPHOS, and FAO are predominant pathways linked to metabolic changes in mitochondria under pathological circumstances. As the key enzyme in the TCA cycle, the alpha-ketoglutarate dehydrogenase complex (KGDHC) showed a 44% reduction in the activity in familial AD patient brain samples,^178^ and KGDHC immunostaining in PD patient tissues showed a reduction in the number of melanized neurons.^179^ Subsequent research showed that the loss of KGDHC-enriched cells was proportional to the total loss of neurons.^180^ Other TCA core enzymes, such as pyruvate dehydrogenase (PDH) and isocitrate dehydrogenase-1 (IDH1), have been analyzed in subsequent clinical studies,^181,182^ and the results suggested TCA enzymes were functional in the pathological brain. In addition, the OXPHOS complex was suppressed under neurodegenerative conditions,^183–185^ indicating lower mitochondrial oxidative respiration efficiency and increased ROS production, both of which impair neurons.^186–188^ A transgenic AD mouse model showed decreased expression of NADH:ubiquinone oxidoreductase subunit B8 (NDUFB8), which is a nuclear DNA-encoded subunit that is integral to the assembly of Complex I.^189^ Further research revealed that NDUFB8 protein O-GlcNAcylation impairment was involved in the high-fat diet (HFD)-induced neurodegenerative process.^190^ Similarly, other respiratory chain proteins, such as oligomycin sensitivity-conferring protein (OSCP), physically interacts with amyloid beta (Aβ) in the brains of AD individuals and AD mouse models.^191^ Moreover, restored mitochondrial bioenergetics enhanced cognition in an AD (5xFAD) mouse model by blocking the deleterious impact of CypD on OSCP or by overexpressing OSCP.^192,193^ Increased binding among Aβ, the apoE4 fragment, Tau and respiratory chain proteins leads to mitochondrial dysfunction.^194,195^ Complex I activity was disrupted during OXPHOS by Ndufs2 deletion and caused human-like PD in mice.^196^ These studies suggest a correlation between mitochondrial respiratory chain proteins and the progression of brain disease. Regarding mitochondrial FAO adaptation in brain pathology, both AD and PD models showed a systemic shift from glycolysis to lipid metabolism,^197,198^ and the expression of mitochondrial lipid metabolism-related proteins was elevated in AD and PD models.^197,199^ Fatty acid metabolism was also upregulated in both pathologies to compensate for neuronal glucose hypometabolism. The carnitine palmitoyl transferase (CPT) system is crucial for mitochondrial β-oxidation of long-chain fatty acids (Table 3).^200^ Overexpression of CPT1c in the mouse brain caused microencephaly, and CPT1c-KO mice demonstrated a marked reduction in spatial learning ability^201,202^ and increased sensitivity to oxidative stress.^203^ In addition, genetic or pharmacological inhibition of acetyl-CoA acetyltransferase 1 (ACAT), an enzyme that catalyzes the final step in the mitochondrial beta-oxidation pathway, is thought to exert an inhibitory effect on the brain lesion process.^204–207^

In addition to the removal of damaged mitochondria in neuronal cells via fusion and fission and mitophagy to maintain mitochondrial homeostasis, the mitochondrial protein unfolded response (UPR^mt^), which is involved in the maintenance of mitochondrial function, is becoming better understood. A transcriptome analysis of the prefrontal cortex of AD patients revealed that UPR^mt^-related genes (HSPA9, HSP60, and YMEL1L) were upregulated, while genetic or chemical inhibition of HSPA9 strongly induced mitochondrial fragmentation and synergistically increased Aβ-mediated cytotoxicity as well as mitochondrial dysfunction.^208^ UPR^mt^ is a conserved mitochondrial stress response signature in diseases involving Aβ proteotoxicity in both humans and mice.^209^

### The cardiovascular system

The cardiovascular system is a vital organ system that delivers essential substances to all cells to support basic functions. This network is composed of the heart, the centralized pump; blood vessels that distribute blood throughout the body; and blood, which transports different substances. Cardiovascular homeostasis relies heavily on mitochondrial fatty acid-driven oxidative phosphorylation for ATP production.^210^ Cardiovascular disease, including myocardial infarction, and cardiomyopathies of different etiologies, including forms of arrhythmia, hypertension, atherosclerosis, and other vascular conditions, is the main “killer” in humans,^211^ and mitochondrial dysfunction is a central etiological determinant of cardiovascular disease.^212^ Strong evidence has shown mitochondrial morphological heterogeneity in several cardiovascular diseases,^213^ and the interchange of the mitochondrial morphology between elongated interconnected mitochondrial networks and a fragmented disconnected arrangement has been found to be relevant in various aspects of cardiovascular diseases. Recently, multiomics studies of cardiovascular disease have demonstrated a role for mitochondrial component heterogeneity in the cardiovascular system, specifically suggesting that increased mitochondrial heterogeneity plays a role in cardiovascular pathology (Table 2). Genome sequencing has led to the identification of mitochondrial gene mutations, including mitochondrial genomic tRNA and respiratory chain coding gene mutants that are linked to cardiovascular diseases.^214,215^ Systematic studies have described the pleiotropic effects of different mtDNA variants and identified novel associations between these variants and previously uncharacterized complex and quantitative traits.^44^ Moreover, high-throughput proteomic and metabolomic analysis of a 2-week ventricular-tachypaced congestive heart failure dog model revealed significant myocardial mitochondrial alterations, particularly the downregulation of oxidant proteins (superoxide dismutase (SOD) and peroxiredoxin (PRX-3)) and the upregulation of TCA core enzymes (malate dehydrogenase (DH), α-/β-enolase (ENO1 and ENO3) and pyruvate dehydrogenase (PDHA1)).^216^ Global proteomic surveys of cardiac ventricles isolated from failing human hearts led to the identification of 25 proteins with significantly changed expression, with 7 proteins located in mitochondria and associated with metabolism, antioxidant activity and the UPR^mt^ ^217^ Therefore, cardiovascular metabolic derangements, oxidant clearance and proteostasis contribute to cardiovascular pathogenesis.

Myocardial metabolic disorders are largely affected by the TCA cycle, OXPHOS and FAO. During pathological heart remodeling, cardiac metabolism is reprogrammed to increase reliance on glucose and significantly increase glycolysis, whereas OXPHOS and FAO are downregulated.^28^ Increased glycolysis is associated with the uncoupling of OXPHOS, resulting in increased lactate production and inhibition of the branched chain amino acid (BCAA) degradation pathway via downregulated KLF15, which promotes a hypertrophic response in cardiomyocytes.^218,219^ All these factors reduce the efficiency of ATP synthesis and exacerbate pathological remodeling.^219,220^ Mitochondrial metabolism adaptation associated with early cardiac pathology contributes to the pathogenesis of heart failure (HF). During the progression from compensated cardiac hypertrophy to HF, net mitochondrial protein acetylation increases, and the acetylation of some of these proteins, such as succinate dehydrogenase complex. subunit A (SDHA), decreases their catalytic function, suggesting that mitochondrial protein acetylation homeostasis is a potential driver of the development of the energy metabolism dysregulation that contributes to heart failure.^135^ One-half of the acetylation sites have been identified previously as potential targets of sirtuin 3 (SIRT3) deacetylase activity in the mouse heart.^166^ Other TCA cycle enzymes (ACO2, IDH2, and MDH2) have also been shown to be acetylated, and heart-specific knockout or overexpression of TCA-associated enzymes in mice demonstrated exacerbated or attenuated the acquisition of cardiac pathological phenotypes (Table 3). Cardiac PDH E1a deficiency caused a large myocardial infarct area and increased macrophage infiltration in the heart, while PDH activated by dichloroacetate in WT hearts during ischemia/reperfusion increased glucose oxidation and reduced myocardial infarct size.^221^ Moreover, in an IDH2-deficient mouse model, mitochondrial dysfunction and cardiac hypertrophy were promoted by PDH activation.^222^ Mitochondrial long-chain fatty acid (LCFA) oxidation, the main FAO pathway involved in myocardial energy supply, was also inhibited due to the reduced activity of the rate-limiting enzyme CPT1. Heterozygous CPT1-knockout mice subjected to the transverse aorta constriction exhibited exacerbated cardiac hypertrophy and remodeling.^223^ Heart-specific (3OH-Butyrate dehydrogenase, type1) BDH1-overexpressing transgenic mice were resistant to fibrosis, contractile dysfunction, and oxidative damage, suggesting that increased ketone body utilization decreased oxidative stress and protected against HF.^224^ Hence, targeting key enzymes of mitochondrial FAO is important for the treatment of heart disease.

ROS accumulation can damage cellular lipids, proteins, and DNA, and a pioneering study revealed that mitochondrial antioxidant proteins (SOD and PRX-3) were downregulated in an HF animal model. A mitochondrion-located redox enzyme (PRX-3) tissue-specific knockout mouse model showed impaired antioxidant capacity and exacerbated cardiac dysfunction and oxidative stress during HF.^225^ Cardiomyocyte-specific SOD2-deficient mice die at ~4 months due to HF and showed mitochondrial architecture alterations, with prominent disruption of cristae and increased vacuole formation.^226^ Another novel mechanism related to the ROS clearance pathway has also been reported. Research has suggested that nicotinamide nucleotide transhydrogenase (Nnt) mediates a reverse reaction in which NADPH is consumed to support NADH and ATP production under pathological heart conditions, which results in a reduction in NADPH-linked antioxidative capacity,^227^ Inhibition of Nnt led to the reversal of its catalytic function in a mouse model and protected against oxidative stress, HF, and death.^227^

### The liver

The liver is the hub of intermediary metabolism supporting key anabolic pathways that synthesize glucose, lipids, and ketones to carefully meet the energy requirements of peripheral tissues.^29^ A study revealed that the primary hepatic mitochondrial structure showed mostly discrete globular or short tubular mitochondria,^228^ while the mitochondrial morphology of primary hepatocytes isolated from rats continuously fed ethanol showed increased heterogeneity.^228^ In a mouse model carrying mtDNA point mutations, elongated mitochondrial networks with an artificial loop structure, depressed autophagy, high mitochondrial respiration and an upregulated antioxidative response were found in liver tissue sections and isolated hepatocytes, which indicated that mtDNA mutations accelerated liver ballooning and degeneration.^229^ Next-generation sequencing (NGS) applied to hepatitis B virus (HBV)-related hepatocellular carcinoma patients revealed that patients with D-loop mutations in mtDNA were more likely to undergo relapse.^230^ In addition, mitochondrial genome nucleotide polymorphism sites were identified in genes related to nonalcoholic fatty liver disease (NAFLD) development.^231^ Multiomics has suggested that metabolic remodeling plays key role in liver pathogenesis.^232,233^ Mitochondrial proteome sequencing of mouse acute liver injury, NAFLD or liver fibroblasts showed the high plasticity of mitochondrial proteins (Table 2).

Acetaminophen (APAP) is a widely used analgesic and antipyretic drug, the overdose of which causes severe centrilobular hepatic necrosis in humans and experimental animals. Recent studies showed that the expression of chaperone proteins HSP10 and HSP60 and glutathione (GSH) was reduced in mitochondria due to treatment of APAP at toxic doses in mice that had been fasted overnight.^234^ The potential therapeutic benefits of GSH, HSP10 and HSP60 have been described in liver disease.^235^ A mechanism explaining the decrease in GSH content during liver disease first involves a decrease in the GSH biosynthesis rate. Then, hepatocyte-specific knockout of glutamate cysteine ligase catalytic (GCLC) protein, the catalytic subunit of the rate-limiting and regulatory enzyme glutamate cysteine ligase (GCL) in the GSH biosynthetic pathway, showed marked mitochondrial morphology changes and a profound decrease in ATP generation in conjunction with histological features of hepatic steatosis.^236^ Livers from GCLC-specific-knockout mice developed spontaneous liver pathologies characteristic of various clinical stages of liver injury.^236,237^ In addition to the de novo GSH synthesis pathway, GSH is found in both the reduced (GSH) and oxidized states (GSSG), and the reduction in GSSG with a commensurate increase in GSH mediated by the enzyme GSH reductase aids in maintaining the GSH level.^238^ Both the reduction and oxidation pathways require NADPH to provide reducing equivalents, and high IDH1 expression in the liver is an important source of cytosolic NADPH. Studies have identified that IDH1-knockout mice were more sensitive to LPS-induced sepsis, which was attributed to a large increase in the hepatocyte apoptosis rate.^239^ Although liver-specific HSP10- and HSP60- knockout mouse models have not yet been reported, research has revealed that chlorogenic acid, liquiritigenin and liquiritin alleviated hepatic inflammatory injury by inhibiting HSP60 release.^240,241^ Interestingly, 75-kDA glucose-regulated protein (GRP75) is a major component of both the mitochondrial quality control system and mitochondria-associated membrane,^242^ and overexpression of GRP75 in the liver decreased cytochrome c expression in CCL4-induced liver injury.^243^

Further studies revealed that the regulation of hepatic mitochondrial proteins at the molecular level during liver injury and disease is associated with mitochondrial metabolism (TCA cycle, OXPHOS, FAO, and ketogenesis), ROS and protein stabilization (Table 3). Studies on liver mitochondria isolated from a rat model of spontaneous diabetes revealed that the levels of FAO and OXPHOS proteins were increased after the rats were rendered diabetic, while the levels of ROS-detoxifying enzymes were decreased.^244,245^ Further study revealed reversible phosphorylation that was widespread in mitochondrial proteins related to OXPHOS, the TCA cycle, FAO, the urea cycle, hormone metabolism, and glycolipid biosynthesis,^133^ with the enzymes involved in ketogenesis (Hmgcs2), lipogenesis (Gpam), and retinol metabolism (Dhrs4) the most significantly changed via modification.^133^ A key mitochondrial enzyme in ketogenesis, 3-hydroxy-3-methylglutaryl CoA synthase 2 (HMGCS2), showed the greatest change in phosphorylation,^133^ indicating that phosphorylation of HMGCS2 plays a key role in the regulation of liver mitochondrial ketogenesis metabolism. Patients with NASH presented with significant increases in hepatic mitochondrial FAO,^246^ the TCA cycle,^247^ OXPHOS^245^ and ketogenesis,^232^ and both mice and humans under chronic nutritional overload showed the impaired mitochondrial function of the TCA cycle,^232^ OXPHOS^232,248^ and ketogenesis.^249,250^ Thus, mitochondrial metabolic function is upregulated in NASH to accommodate the rapid accumulation of hepatic triglycerides. In an NAFLD model, fat extracted by the liver coupled with mismatched mitochondrial fat disposal capacity led to fat accumulation in hepatocytes. A study suggested that the accumulation of plasma triglycerides derived from the liver and dietary free fatty acids (FFAs) and de novo lipogenesis in simple steatosis are associated with mitochondrial metabolism adaptation.^246,247,251^ This adaptation of mitochondrial metabolism is the central feature of NAFLD.^248,252^

In addition, mice fed an HFD showed normal mitochondrial energetics but accumulated lipotoxic byproducts, including ceramides and diacylglycerols. This finding highlights the complex interaction between early compensatory oxidative mechanisms and the inefficient storage/disposal of FFAs.^233^ The mitochondrial FAO pathway regulation mechanism plays an important role in liver pathology progression, and studies have shown that CPT1α, CPT2α, ACAT and ACAC/ACC are core regulatory sites.^253^ Permanently active CPT1 mutants enhanced hepatic FAO and autophagy, reduced liver steatosis, and improved glucose homeostasis in HFD mice, suggesting that CPT1 gene therapy reduced HFD-induced dysregulation.^254^ To further illustrate the interactions between lipid metabolism and other cellular metabolic functions, a systematic analysis was performed with hepatocytes to distinguish NASH-specific metabolic features. This study identified PHGDH, SHMT1 and SHMT2 as potential mitochondrion-targeted therapeutic options for NASH and showed that downregulation of these proteins resulted in serine synthesis pathway blockade.^255^ In addition to the fat-processing burden placed on the hepatic mitochondria, a high ROS burden has been associated with liver pathology. In high hepatic cholesterol-induced NASH and fibrosis, mitochondrial proteins related to ROS generation, such as cytochrome P450-2E1 (CYP2E), were upregulated,^256,257^ and Cyp2e1-null mice showed resistance to high cholesterol-induced NASH and fibrosis,^256^ while ROS clearance proteins, including SOD1, SOD2, Gclc, Gclm and Gpx1, were downregulated.^258–262^ Silibinin is used for the clinical treatment of NASH because it significantly activates antioxidase activity (CAT, GSH-Px and HO-1) and inhibits pro-oxidase activity (CYP2E1 and CYP4A) to reduce ROS generation.^257^

### The skeletal muscle

The influence of skeletal muscle, which accounts for as much as 40% of body mass, has multiple implications for mobility, injury, and metabolic diseases and thus exerts a major impact on overall quality of life^263^. Skeletal muscle plays a prominent role in metabolic homeostasis and is closely associated with mitochondrial oxidative metabolism^263^. Mitochondria form a reticulum within muscle cells and are classified into subsarcolemmal (SS) mitochondria and intermyofibrillar (IMF) mitochondrial types.^264^ SS mitochondria exhibit a circular morphology, while IMF mitochondria exhibit a long and branched morphology.^265^ Mitochondrial morphology heterogeneity has been observed in a mouse skeletal muscle aging model, suggesting that mitochondrial morphology heterogeneity is associated with skeletal muscle pathology.^265^

Muscle mitochondrial metabolism diversity depends on different types of muscle fibers^266–268^ and different muscle pathology conditions (obesity, type 2 diabetes (T2D), and aging)^267,269,270^ (Table 2). In 1991, a male patient with myopathy and neuropathy presented with large-scale deletion of the mitochondrial genome at nucleotides 6570-14150.^271^ Mitochondrial genome mutations and large-scale deletions were also found in Leigh syndrome and myopathy.^272^ Further study identified two new point mutations, A189G and T408A, which had accumulation in muscle tissue, but not in other tissues, of several older individuals.^273^ This study revealed a close association between mtDNA mutations and specific mutagenic machinery.

The SS and IMF mitochondrial subpopulations indicate different susceptibility to obesity, with IMF mitochondria showing upregulated TCA cycle enzyme activity but downregulated OXPHOS protein activity, in contrast to SS mitochondria.^274^ Furthermore, skeletal muscle consists of three major fiber types: slow oxidative Type 1 fibers, fast oxidative Type 2a fibers, and fast glycolytic Type 2x fibers.^275^ Characterization of the proteome of isolated single fibers from the extensor digitorum longus muscle in mice revealed that the abundance of proteins involved in OXPHOS, FAO, and the TCA cycle varied between the individual muscle fiber types, with Type 2a and Type 1 fibers showing a greater abundance of OXPHOS and FAO proteins.^276^ A proteome analysis of the skeletal muscle of patients with T2D taken by biopsy led to the identification of ATP synthase β-subunit (ATP5F1B) downregulation. Moreover, ATP5F1B was phosphorylated in vivo, and the levels of a downregulated ATP5F1B phospho-isoform in diabetic muscle correlated inversely with fasting plasma glucose levels.^277^ A subsequent study on mitochondrial OXPHOS protein phosphorylation reported that abnormal site-specific phosphorylation of ATP5F1B, together with reduced OXPHOS protein content, contributed to mitochondrial dysfunction during muscle insulin resistance.^278^ Recent studies have even described an accurate mitochondrial protein atlas of T2D pathological states. A comparison of the mitochondrial proteomes between T2D and nondiabetic skeletal muscle samples identified mitochondrial functions (OXPHOS, TCA, FAO, and the ROS response) related to T2D.^279^ Thus, muscle mitochondrial proteomic studies provide guidance and direction for studying the regulatory mechanisms of mitochondrial protein content and modifications in T2D.

Aging-associated mitochondrial function decline contributes to insulin resistance in elderly individuals,^280^ suggesting that increases in intramyocellular fatty acid metabolites may be results of an age-associated reduction in mitochondrial oxidative and phosphorylation activity.^280^ This result was confirmed via a quantitative proteomic analysis of skeletal muscle collected from young and elderly individuals.^269^ Of the mitochondrial proteins identified, the levels of 173 mitochondrial proteins were changed with age, and these proteins were related to OXPHOS, the TCA cycle and mitochondrial homeostasis. Interestingly, this change in skeletal muscle mitochondrial protein level was partially reversed by physical activity.^281,282^ Myosteatosis is the pathological accumulation of lipids that can occur in conjunction with atrophy and fibrosis following skeletal muscle injury. A pioneering study determined that mitochondrial dysfunction leads to the accumulation of lipids in myosteatosis.^283^ Research evaluating changes during muscle fiber force production showed that mitochondrial FAO was reduced in the early injury process and that the levels of glycolytic metabolites in muscles generally increased and that these metabolites showed a greater capacity to oxidize pyruvate at later points.^283^

### The adipose tissue

Whole-body adipose tissue content and type are controlled in response to various internal and external cues (e.g., nutritional status and temperature).^284,285^ The regulatory processes involved in fat storage and oxidation in white adipocytes and thermogenic adipocytes (brown and beige adipocytes) play central roles in body energy homeostasis,^286,287^ and adipose tissue mitochondrial dysfunction in pathological states such as obesity, insulin resistance, and chronic inflammation is closely associated with adipose malfunction.^288–292^ Indeed, BAT and beige adipocytes exhibit fragmented round-shaped mitochondria, while white adipocytes exhibit elongated organelles with high levels of ATP synthesis.^293^ This mitochondrial morphology heterogeneity can determine uncoupling protein 1 (UCP1) content, suggesting that mitochondrial morphology is associated with thermogenesis.^293^ Increased somatic mtDNA mutations resulting from POLG mutation is associated with a reduced lifespan and premature onset of aging-related phenotypes, such as weight loss and reduced subcutaneous fat.^294^ Mitochondrial thymidine kinase 2 (Tk2) is vital to maintaining appropriate levels of mtDNA. A mouse model with null mutation of Tk2 showed mtDNA depletion, moderate hypotrophy in adipose tissues, and reduced fat accumulation.^295^ Moreover, overexpression of mitochondrial targeted 8-oxoguanine DNA glycosylase, which has been associated with the base-excision repair pathway, protected against diet-induced obesity, insulin resistance, and adipose tissue inflammation.^296^

Mitochondrial proteomics has revealed the relevance of mitochondria in adipose tissue and the systemic implications of their impaired function. WAT mitochondria not only selectively express proteins that support anabolic functions but also degrade xenobiotics, while BAT mitochondrial proteins are particularly suited to catabolic functioning.^297^ Adipose tissue plays important pathophysiological roles in metabolic abnormalities, such as obesity, T2DM and aging. A proteomic analysis of visceral adipose tissue (VAT) in early T2DM patients and control individuals showed downregulation of the TCA cycle, FAO and OXPHOS, while the mitochondrion-related ROS response was upregulated.^289,298^ Interestingly, aging led to a change similar to that of T2D pathology with mitochondrial remodeling,^291,299^ indicating that the mitochondrial proteome plays an important role in the aging-related pathology of T2D.^300^ BAT mitochondria dissipate chemical energy as heat through thermogenic respiration, which requires UCP1.^301–305^ Studies have revealed that UCP1 C253 sulfenylation^302^ and K56/K151 hypersuccinylation^304^ play important roles in UCP1-dependent thermogenesis and whole-body energy expenditure. Further study found significant inhibition of thermogenic responses in UCP1-C253A-mutant mice.^305^ UCP1-dependent thermogenesis in adipose tissue plays an important role in obesity. Furthermore, quantitative mitochondrial proteomics of BAT and beige adipose tissue has led to the identification of arginine/creatine metabolism as a beige adipose signature induced in response to cold exposure.^301^

Although a recent study identified other UCP1-independent thermogenic mechanisms,^303^ mitochondrial TCA metabolism and pyruvate dehydrogenase activity are associated with ATP-dependent thermogenesis, suggesting that mitochondrial thermogenesis function plays an important role in BAT and beige adipose function. An increasing number of mitochondrial proteins have been identified in adipose pathologies, such as OXPHOS proteins (Ndufv2 and Ndufs4)^306,307^ and ROS-response proteins (MnSOD).^308^ A study identified that overexpression of Ndufv2 in adipose tissue mediated increases in mitochondrial biogenesis by regulating supercomplex assembly and elevating mitochondrial ROS production.^306^ Genetic deletion of Ndufs4 in adipose tissue resulted in an increased propensity to develop diet-induced weight gain, glucose intolerance, and elevated levels of fat-related inflammatory genes, specifically in young male mice. Both studies linked mouse adipose phenotypes to the mitochondrial respiratory chain and identified sex differences at the genetic level.^306,307^

### The immune system

The immune system is important in protecting against infection and cancer. Studies have focused on mitochondrial functions in immune cells.^309^ The immune system consists of the innate immune system and the adaptive immune system. Innate immune response cells include monocytes, macrophages/dendritic cells, granulocytes (neutrophils, eosinophils, and basophils), and innate lymphocytic cells, including natural killer (NK) cells. The adaptive immune system consists of T and B lymphocytes.^309^ These cells recognize a foreign agent and mount an inflammatory response, and previous works have revealed that mitochondria are rapidly reprogrammed to meet the demands of effective immune responses.^310^ Notably, NK cell mitochondria exhibited a small spherical mitochondrial shape, while NK cells infected by human immunodeficiency virus exhibited a long and tubular mitochondrial morphology. The discovery of mitochondrial heterogeneity in immune cells has also been reproduced in macrophages, monocytes and T lymphocytes.^311,312^ Mitochondrial genome sequencing has been applied to immune cells. A pioneering study showed that the mtAtp8 polymorphism increased the adaptive potential of CD4^+^ T cells when OXPHOS was impaired.^313^ Further study of the mtAtp8 (m.7778G>T) polymorphism in CD4^+^ T cells showed a differential cellular respiration profile that led to modified cytokine production in the CD4^+^ T cells.^314^ These observations showed that mtDNA mutations affected the immune system, but immune cells still maintained proper functioning despite their high mtDNA mutation load. One possible mechanism may involve mtDNA replication that lags cell proliferation, which is evident in both pro-B and pre-B progenitor cells, because it reduces the number of mtDNA copies per cell and causes a genetic bottleneck.^315^

Mitochondrial proteomics fuels the study of mitochondrial adaptive changes during the immune response. Mitochondrial proteomics has highlighted the close association between mitochondrial energy metabolism and the innate immune response. Monocytes are key inflammation coordinators and act as direct effectors of innate immunity. A previous study explored the proteome of monocytes in sepsis and revealed that glycolytic proteins showed consistent positive regulation, while TCA and OXPHOS were negatively regulated in the sepsis group, and these differences were largely reversed in the recovery group.^316^ Macrophages play an important role in pathogen elimination via phagocytosis, in which pathogens are deactivated by the gradual acidification of the phagosome and exposure to mitochondrion-derived ROS.^317,318^ A recent study on macrophage mitochondria in bacterial infections suggested that OXPHOS was negatively regulated, consistent with increased mitochondrial ROS generation.^319^ The mitochondrial proteome has also been assessed in adaptive immune cells, such as in T cells during activation.^31,320,321^ A pioneering study showed that naïve CD4^+^ T-cell activation induced a unique program of mitochondrial one-carbon metabolism.^31^ Supplementing cell cultures with exogenous serine and inhibition of the mitochondrial serine catabolic enzyme SHMT2 illustrated the critical role of the mitochondrial one-carbon metabolism pathway in T-cell activation and survival.^31^ In particular, the activation of aged naïve T cells was enhanced by the addition of products of one-carbon metabolism (formate and glycine).^321^ Other proteomic research on T cells has also suggested that mitochondria are critical for immune function. Systemic reconstruction of regulatory networks underlying T-cell activation led to the identification of mitochondrial pathways, including mitoribosomes and Complex IV-mediated OXPHOS. T-cell COX10-specific-knockout mice showed greatly elevated cell death rates and impaired cell proliferation.^320^ In addition to the mitochondrial proteomic study of immune cells, pharmacological and genetic inhibition of other mitochondrial proteins has yielded a broad overview of immune responses.^322,323^ Acsbg1, a member of the ACSL family, was selectively expressed in Treg cells, and genetic deletion of Acsbg1 not only caused mitochondrial dysfunction but also dampened other metabolic pathways.^322^ Furthermore, genetic deletion of mitochondrial pyruvate carrier (MPC) drove CD8^+^ T-cell differentiation toward the acquisition of a memory T-cell phenotype due to increasing glutamine levels and FAO. In contrast, short-term inhibition of MPC in activated T cells enhanced antitumor activity.^323^

### Mitochondrial heterogeneity in cancer

Cancer involves the rapid proliferation of abnormal cells that accumulate into tumors that grow beyond their usual boundaries and can spread to other organs via metastasis.^324^ Cancer heterogeneity clearly contributes to the plasticity of cancer cells.^325^ A corollary to this view is that cancer plasticity is mainly affected by the tumor microenvironment via epigenetic modification,^326,327^ while increasing evidence tends to support the importance of mitochondrial heterogeneity in cancer heterogeneity.^328,329^ Accumulation of mtDNA mutations during aging has been reported in various tumor types, such as kidney, colorectal and thyroid cancers.^330,331^ More than 85% of mtDNA obtained from 21 cancer tissues and 38 cancer types carried mutations, with the ND5 gene located in mtDNA the most frequently mutated in most cancer cells and with ND4 frequently mutated in prostate and lung cancers.^330^ Sequencing of the mtDNA mutation in tumor epithelial cells demonstrated that 44% of mtDNA mutations in adenomas and 85% of mtDNA mutations in adenocarcinomas were tumor specific. Moreover, these mutations have also been observed in the mtDNA of normal crypts, suggesting that mtDNA heterogeneity in normal crypts may provide a selective metabolic advantage during tumorigenesis.^331^

**Fig. 7:**
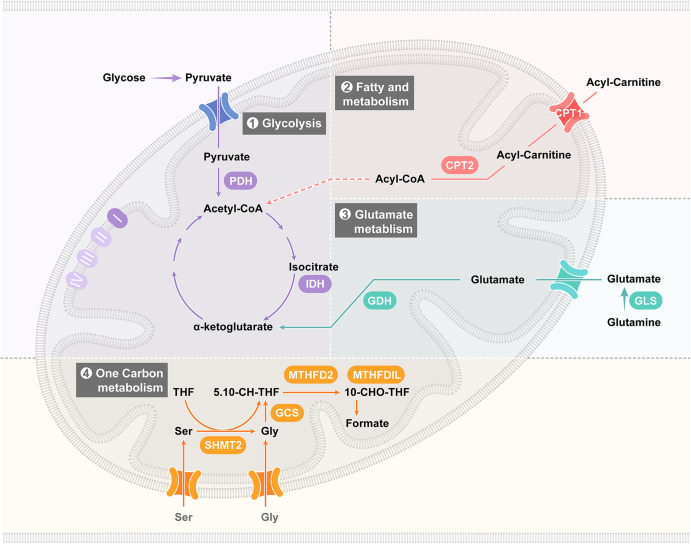
Mitochondrial heterogeneity in cancer. Glycolysis, a potential protein target of key TCA cycle enzymes, is in purple. Fatty acid metabolism, a potential protein target of FAO key enzymes, is in red. Glutamate metabolism, a potential protein target of glutamine metabolism, is in orange. One-carbon metabolism, potential protein targets of one-carbon metabolism are in orange. Abbreviations: PDH pyruvate dehydrogenase, IDH isocitrate dehydrogenase, CPT1 carnitine palmitoyltransferase 1, CPT2 carnitine palmitoyltransferase 2, GLS glutaminase enzyme, GDH glutamate dehydrogenase, SHMT2 serine hydroxymethyltransferase, 2, GCS glycine cleavage system, MTHFD2 methylenetetrahydrofolate dehydrogenase, and MTHFD1L methylenetetrahydrofolate dehydrogenase 1-like

Studies targeting the mitochondrial proteome have provided solid evidence for cancer mitochondrial heterogeneity (Fig. 7).^332^ Different mitochondrial proteins have been identified between nasopharyngeal carcinoma (NPC) metastatic and nonmetastatic cell sublines, and these proteins, such as PRDX3 and SOD2, have been associated with ROS and redox pathways.^333^ Suppression of mitochondrial PRDX3 in the ROS pathway enhanced the mobility potential of NPC metastatic cancer cells.^333^ Furthermore, as determined via the mitochondrial proteomic analysis of human ovarian cancer (OC) cells compared to drug-resistant cell sublines^334,335^ or human OC tissues, a series of enzyme profiles associated with the TCA and OXPHOS pathways was significantly different in OC cells compared to platinum-resistant cell sublines.^336,337^ Some of these enzymes are crucial in tumor progression and maintenance. For example, IDH is overexpressed in OC tissues and plays roles in growth and proliferation, suggesting that mitochondrial heterogeneity contributes to cancer cell drug resistance.^338^ Intriguingly, therapeutics targeting mitochondrial IDH have been implemented in clinical practice.^338^ Research on the human OC mitochondrial phosphoproteome led to the identification of 48 differentially phosphorylated mitochondrial proteins between OC tissue and paracarcinoma tissue.^339^ The mitochondrial proteome profiling of human OC and NPCs has provided ideal models to study therapeutic targets in various cancers.^332^

A critical metabolic phenotype observed in cancer cells is based on the Warburg effect, in which ATP generation shifts from oxidative phosphorylation to glycolysis, even under normal oxygen concentrations.^340^ It is a long-held view that this cancer cell metabolic reprogramming arises from mitochondrial defects that inhibit the ability to oxidize glucose carbon effectively into carbon dioxide. However, recent research has revealed that most mitochondria in cancer cells are reprogrammed, not subject to functional defects, to function as biosynthetic organelles,^341^ which play critical roles in both tumorigenesis and metastasis.^328,342,343^ Regarding tumorigenesis, heterogeneity of the SDH subunit has been observed in hereditary paragangliomas and pheochromocytomas, and SDH defects account for 10–70% of inherited paragangliomas and 10–30% of pheochromocytomas.^344^ Inhibition of SDH causes the loss of mitochondrial Complex II activity and increased production of mitochondrial ROS that result in the high expression of angiogenic factor in paraganglioma.^345^ In multiple cutaneous and uterine leiomyomata and in aggressive forms of renal cell cancer, fumarate hydratase (FH) heterogeneity is critical to tumor formation.^346^ Because FH is a vital enzyme of the TCA cycle, the heterogeneity of FH in renal cancer cells causes a metabolic shift from the TCA cycle to a linear metabolic pathway beginning with glutamine intake and ending with bilirubin excretion in mitochondria.^346^

In addition, increased mitochondrial redox activities in cancer have been correlated with cancer metastasis.^347^ Higher mitochondrial membrane potential has been associated with cancer cell survival and invasiveness.^348–350^ Recent research has revealed that mitochondrial heterogeneity potentiates cancer cell dissemination and metastasis.^329,342^ In the same host with breast cancer, circulating cancer cells exhibited enhanced mitochondrial biogenesis and respiration compared to cancer cells in the primary tumors,^351^ and heterogeneity in mitochondrial PHGDH protein promoted breast cancer metastasis.^329^ In other models of cancer metastasis, such as oral squamous cell carcinoma and melanoma, invading leader cells have been shown to be associated with an increase in mitochondrial membrane potential and translation rate.^342^

## Therapy targeting mitochondrial heterogeneity

Mitochondrial heterogeneity has been under-diagnosed in clinical practice because of insufficient understanding about the mechanism of the mitochondrial abnormalities underlying various disease pathologic condition. The situation makes it a challenge to develop effective therapies. At yet, there is no clinical treatment for the loss-of-function mutation in the mitochondrial genes, but clinically-approved therapies are available to alleviate the symptoms.^352,353^ Based on the rationale that mitochondrial dysfunction from sustained damage to the organelle’s DNA, proteins and lipids, treatment strategies aimed at oxidative damage, carbon stress and mutation of mitochondrial genes are discussed.

### Therapies targeting mitochondrial oxidative damage

Tissues and organs that have high metabolic rate have higher possibility to suffer from oxidative stress, for example cardiovascular system and nervous system. Several therapies aimed at mitochondrial oxidative stress have been implemented. The use of antioxidants to prevent ROS-induced damage has emerged as the prime therapy. MitoQ is a mitochondria-targeted antioxidant, and preclinical studies demonstrate that MitoQ reduces myocardium damage and improves cardiac function in different animal models of cardiomyopathy.^354,355^ According to preclinical investigations, CoQ10, a component of ETC, attenuates oxidative stress and cardiomyocyte remodeling in rodents with diabetic cardiomyopathy. Treatment of heart failure patients with CoQ10 reduces the number of adverse cardiovascular events and rates of hospitalization and mortality.^356^ Meanwhile, MitoQ and CoQ10 have been tested in metabolic disease and neurodegeneration disease. MitoQ attenuated weight gain and ameliorates hepatic dysfunction in obese rodents and oxidative stress, synaptic loss and amyloid beta peptide accumulation which is associated with preserved cognitive function in a rodent model of AD. Clinical data suggest that MitoQ, as antioxidant treatment, increase insulin sensitivity in patients with diabetes.^357^ Other attractive compounds such as Bendavia and Cyclosporine A, that reduce ROS release, may have demonstrated clinical referential value.^358,359^ Several studies have revealed that inhibited ROS generation shows different aspects in cardiovascular system, immune system and liver alleviated pathologic conditions. Dimethyl malonate (DMM), inhibitor of SDH, has been decreased succinate accumulation and oxidation during heart ischemia and reperfusion (IR).^360^ Further study has observed DMM to inhibit LPS-induced mtROS generation in inflammatory macrophage and to promote an anti-inflammatory outcome.^361^ While inhibition of SDH in activated T cells has impaired T cell activation and function.^362^ These studies revealed that appropriate clinic treatment of mtROS inhibition should be based on the tissue mitochondrial heterogeneity.

### Therapies targeting mitochondrial carbon stress

Mitochondrial carbon stress exhibits high level of activated acyl-CoA and the depletion of the mitochondrial NAD^+^ pool, which is also manifested in pathogenic conditions, such as liver disease, AD, IR and diabetes. Mitochondrial carbon stress may result in protein acylation, the accumulation of misfolded and damaged proteins, and the disruption of protein function and proteostasis.^352,353^ Clinical therapies targeting mitochondrial carbon stress have also been implemented. A related common pathway of mitochondrial carbon stress is the depletion of NAD^+^. Therefore, many protective effects have been demonstrated in fatty liver disease, AD and diabetes by administrating compounds such as nicotinamide (NAM), NR, and NMN, whose function by replenishing NAD^+^ levels and activating sirtuins is to counteract carbon stress.^209,363,364^ Furthermore, clinical treatments that protect the mitochondria from carbon stress directly by affecting TCA cycle and OXPHOS in pathologic states. As mentioned, SDH, as a target to alleviate pathologic process has been reported in IR and immune response. More TCA cycle enzymes emerged as a target to release carbon stress in pathological conditions have been reported, including PDH and IDH. PDH activated by dichloroacetate in WT heart during IR has been observed to reduce myocardial infarct size. As for the IDH, previous studies have found that IDH-deficient mouse model has shown mitochondrial dysfunction and cardiac hypertrophy.^222^ Moreover, inhibition of complex I by mitochondria-targeted S-nitrosating agent MitoSNO has been used to decrease ROS production and cardiac IR injury.^365^ It is likely that many other agents that act in a similar way against type 2 diabetes. Metformin is widely used for the treatment of type 2 diabetes that inhibits complex I, elevates the ADP:ATP ratio and thereby activates liver AMPK to slow liver gluconeogenesis.^366^ Recent study has revealed that metformin ameliorates acute respiratory distress syndrome by inhibiting complex I through disruption of ATP and mtDNA synthesis.^367^

In addition, compounds that target mitochondria, such as doxycycline (DOX),^368,369^ nicotinamide riboside (NR) and olaparib (AZD2281 or AZD), exhibit the ability to enhance UPRmt.^370^ DOX markedly induces the transcription of UPRmt, increases mitophagy and induces respiration gene expression in GMC101 worms and reduces intracellular Aβ deposits in a human neuroblastoma cell line (SH-SY5Y).^370^ The treatment of NR and AZD to GMC101 worms also induces the UPRmt and improves lifespan, but the treatment of NR and AZD to CL2122 improves the UPRmt only in aging.^370^ These studies provide clinical prospect in targeting unfold protein accumulation.

### Therapies targeting mitochondrial biogenesis and dynamic

Intercellular and intracellular imbalances of mitochondrial biogenesis and distribution cause pathological states in various tissues and organs.^371,372^ This kind of heterogeneity has become a potential target of clinical interventions. Instead of directly affecting mitochondria, more alternative therapeutic strategy is to alter mitochondrial amount by enhancing mitochondrial biogenesis and modulating mitochondrial dynamic by inhibiting the protein machinery. In targeting mitochondrial biogenesis, previous studies have demonstrated many drugs interact with mitochondrial biogenesis pathway by altering the activity of transcription factors. These drugs include anti-diabetic drugs pioglitazone and rosiglitazone as well as by the lipid metabolism modifiers thiazolidinediones which increase PGC1α expression levels and enhance mitochondrial biogenesis.^373–375^ Meanwhile, 5-aminoimidazole-4-carboxamide ribonucleotide (AICAR), agonist directly activating PGC1α, has been demonstrated to enhance mitochondrial biogenesis by activating PGC1α, resulting in a partial repair of mitochondrial myopathy mice model.^376^

Small compounds have been developed to target mitochondrial dynamic. Such as Mdivi1, mitochondrial division inhibitor 1, which decreases DRP1 activity can slows mitochondrial fission.^377^ Previous studies have revealed that Mdivi1 is neuroprotective in neurodegeneration model by inhibiting Drp1-dependent mitochondrial fission.^378^ But in many cases it still remains unclear of the beneficial effect of modulating mitochondrial dynamic. One of the important aspects of modulating mitochondrial dynamic is that they are intimately linked to the mitochondrial quality control. As for compounds that induce mitophagy to maintain mitochondrial quality, the urolithin A (UA) prevents the accumulation of dysfunctional mitochondria with aging, extends lifespan in *C. elegans* and increases muscle function in rodents.^379^ Mitochondrial transfer has been shown to be an attractive prospect that could be a potential therapeutic approach in targeting mitochondrial biogenesis. Previous studies have reported that healthy mitochondria were released from astrocytes into the extracellular space and then were transferred into energetically stressed neurons to maintain neural tissue hemostasis.^380^

### Therapies targeting mitochondrial gene mutation

Correction of mitochondria mutation gene is emerging as a critical intervention approach targeting mitochondrial heterogeneity. In previous studies, mitochondrial-targeted restriction endonucleases or transcription activator-like effector nucleases (TALENs) have been applied to selectively reduced human mutated mtDNA levels responsible for Leber’s hereditary optic neuropathy.^381^ Recently, mitochondrion-targeted zinc-finger nucleases (mtZEN) have been applied to specifically eliminate the deleterious mtDNA m.5024C>T mutation in vivo throughout the mouse heart.^164^ In combination with programmable nucleases and tissue-specific adeno-associated viruses, this mtDNA gene-editing tool may offer a potentially universal route for treating mtDNA heterogeneity-related diseases.^164^ Meanwhile, the mitochondria-targeted meganucleases (mitoARCUS) is equally capable of eliminating mtDNA m.5024C>T mutation throughout the mouse liver and skeleton muscle in vivo, with a relatively small size and the ability to recognize one-base mutation.^382^ Current studies have achieved to catalyze C•G-to-T•A conversions and A•T -to- G•C conversions in human mtDNA with high target specificity and product purity.^163,383^ Research have engineered a interbacterial toxin variant, named DddA variant, that catalyzes the deamination of cytidines within dsDNA until brought together on target DNA.^163^ Fusion of the DddA variant, TALENs and uracil glycosylase inhibitor resulted in RNA-free DddA-derived cytosine base editors (DdCBEs), which catalyzes C•G-to-T•A conversions in human mtDNA, and the application of DdCBEs to disease-associated mtDNA mutations has been shown to induce the recovery of respiration rates and OXPHOS in human cells.^163^ Further studies have made an fusion of DddA variant, TALENs and an engineered deoxyadenosine deaminase to resulting in catalyzing A•T -to- G•C conversion in human mt DNA.^383^ These studies have provided prospect in clinical treatment of disease-causing mtDNA mutation. However, mitochondrial genome editing efficiency is not yet sufficient for application in clinical therapies.^383^ On the other hand, studies have revealed that mitochondrion-targeted genetic editing may induce extensive off-target changes in the nuclear genome,^384^ which might restrict the application of mitochondrial DNA base editors.

## Conclusion

**Fig. 8:**
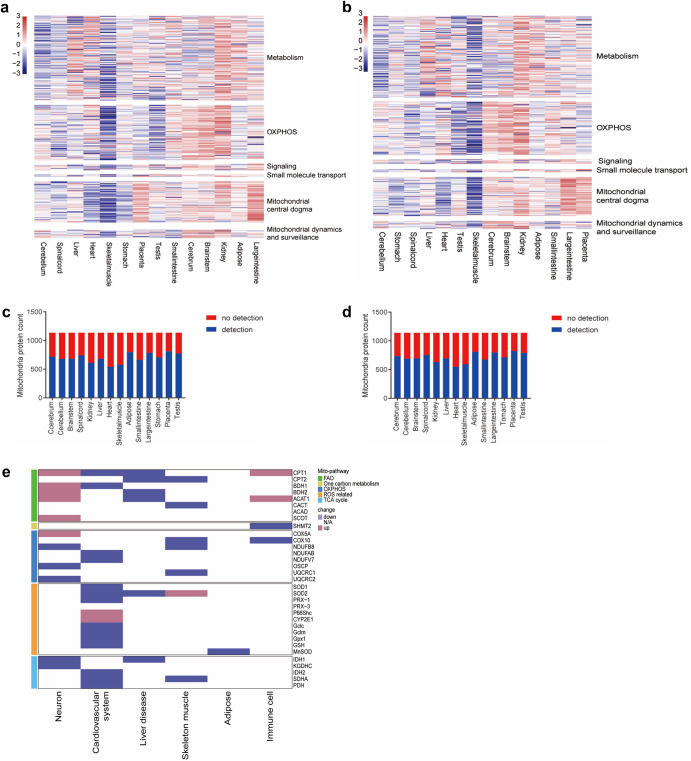
Heterogeneity of mitochondrial proteins in tissues. The mitochondrial protein expression data were obtained from MitoCarta 3.0 (http://www.broadinstitute.org/pubs/MitoCarta). **a** Mitochondrial protein expression in 14 human tissues as determined with MitoPathways. Columns represent different tissues, and rows represent the relative expression levels of different proteins. The R package (pheatmap) was used to draw the heatmap. **b** Mitochondrial protein expression of MitoPathways in 14 mouse tissues. Columns represent different tissues, and rows represent the relative expression levels of different proteins. The R package (pheatmap) was used to draw the heatmap. **c** Number of distinct proteins detected and not detected in 14 human tissues. **d** Number of distinct proteins detected and not detected in 14 mouse tissues. **e** Heatmap showing mitochondrial protein heterogeneity in 6 tissues. Maroon represents upregulated proteins in tissues under pathological conditions, and blue represents downregulated proteins in tissues under pathological conditions

Mitochondrial biology has been extensively studied using traditional biochemical and molecular methods for decades. Technological advances have driven a steep change in our understanding of mitochondrial biology. Systematic studies on mitochondrial multi-omics have led to the discovery of mitochondrial heterogeneity in different cells and tissues (Fig. 8). However, we still know very little about the molecular mechanisms involved, especially the complexity of the mitochondrial genome. A mtDNA phenotype that is neutral in one context may be deleterious in others. Thus, understanding mtDNA-specific heteroplasmic variants’ behavior is extremely important for clinical studies designed to “clear up” pathogenic mtDNA mutations. In the rapidly evolving field of mitochondrial proteomics, the comprehensive characterization of the mitochondrial protein inventory offers exciting opportunities for systematic analysis of this organelle underneath physiological and pathological conditions. Hundreds of mitochondrial proteins that do not appear in the current catalog will likely be identified via a combination of high-throughput and traditional biochemical strategies, resulting in a complete mitochondrial proteome profiling. It will then be important to understand the extent of splicing variants and PTMs of all these proteins, as well as their specific localization in mitochondria. An advanced challenge regarding the mitochondrial proteome is then to understand how mitochondrial proteins function together in pathways and complexes. Using high-throughput methods such as RNAi, protein‒protein interaction mapping and computational prediction, most of the uncharacterized proteins will be annotated.

As the protein inventory and complexes of mitochondria are refined, it will become important to describe their heterogeneity in different tissues, developmental states and diseases. The first step in the current characterization of mitochondrial heterogeneity is the genetic study of rare and common variants of the mitochondrial proteome. Because of next-generation sequencing technologies, large projects, such as the 1000 Genomes Project, will soon catalog the range of normal mitochondrial genomic variants. Additionally, resequencing of individuals with extreme mitochondrial phenotypes may reveal an additional set of high-propensity variants. However, establishing genetic links between specific extreme phenotypes and mitochondrial gene mutations remains very challenging. To study the molecular mechanisms of mitochondrial function adaptation and related alterations in different tissues, developmental states and diseases, it is important to obtain high-resolution mitochondrial proteome data and a method to target mitochondria. However, multi-omics technique also presents some limitations. First, due to the limitation of MS-based sequencing, the mitochondrial proteome might miss some bona fide mitochondrial proteins, especially proteins less than 10 kDa or with unfavorable proteolytic cleavage sites.^49^ Second, it is still a challenge to identify proteins that are in mitochondria under specific conditions.^49^

### Supplementary information

Dataset 1,Dataset 2,Dataset 3, Dataset 4
